# Implementation of the SMART MOVE intervention in primary care: a qualitative study using normalisation process theory

**DOI:** 10.1186/s12875-018-0737-2

**Published:** 2018-05-02

**Authors:** Liam G. Glynn, Fergus Glynn, Monica Casey, Louise Gaffney Wilkinson, Patrick S. Hayes, David Heaney, Andrew W. M. Murphy

**Affiliations:** 1 Graduate Entry Medical School and Health Research Institute, University of Limerick, Limerick, Ireland; 2 Southampton University Medical School, Southampton University, Southampton, UK; 3 Discipline of General Practice, National University of Ireland, Galway, Ireland; 4 Centre for Rural Health, University of Aberdeen, Inverness, Scotland

**Keywords:** Exercise, Technology, Health behaviour, Qualitative research, Barriers, Facilitators

## Abstract

**Background:**

Problematic translational gaps continue to exist between demonstrating the positive impact of healthcare interventions in research settings and their implementation into routine daily practice. The aim of this qualitative evaluation of the SMART MOVE trial was to conduct a theoretically informed analysis, using normalisation process theory, of the potential barriers and levers to the implementation of a mhealth intervention to promote physical activity in primary care.

**Methods:**

The study took place in the West of Ireland with recruitment in the community from the Clare Primary Care Network. SMART MOVE trial participants and the staff from four primary care centres were invited to take part and all agreed to do so. A qualitative methodology with a combination of focus groups (general practitioners, practice nurses and non-clinical staff from four separate primary care centres, *n* = 14) and individual semi-structured interviews (intervention and control SMART MOVE trial participants, *n* = 4) with purposeful sampling utilising the principles of Framework Analysis was utilised. The Normalisation Process Theory was used to develop the topic guide for the interviews and also informed the data analysis process.

**Results:**

Four themes emerged from the analysis**:** personal and professional exercise strategies; roles and responsibilities to support active engagement; utilisation challenges; and evaluation, adoption and adherence. It was evident that introducing a new healthcare intervention demands a comprehensive evaluation of the intervention itself and also the environment in which it is to operate. Despite certain obstacles, the opportunity exists for the successful implementation of a novel healthcare intervention that addresses a hitherto unresolved healthcare need, provided that the intervention has strong usability attributes for both disseminators and target users and coheres strongly with the core objectives and culture of the health care environment in which it is to operate.

**Conclusion:**

We carried out a theoretical analysis of stakeholder informed barriers and levers to the implementation of a novel exercise promotion tool in the Irish primary care setting. We believe that this process amplifies the implementation potential of such an intervention in primary care. The SMART MOVE trial is registered at Current Controlled Trials (ISRCTN99944116; Date of registration: 1st August 2012).

## Background

‘The current literature demonstrates that there are problematic translational gaps continue to exist between demonstrating the positive impact of a novel healthcare intervention in a research environment and the implementation of this intervention into routine daily practice [1–4]. Implementation science has emerged precipitately as an increasingly influential research field [5] by recognizing that the findings from clinical and health services research have often failed to change population health outcomes. [6] The relative failure to implement what we know mirrors the important imbalance between investment in the development of new drugs and medical technologies versus addressing the fidelity with which care is delivered [5]. We have the evidence that interventions can be effective but high quality evidence with regard to the choice and means of optimizing the elements of complex interventions has been much slower to emerge [7].

A greater role for theoretical approaches in implementation-focused research is one proposed strategy for overcoming translation gaps [5]. The Normalisation Process Theory (NPT) is one such theory which furnishes researchers with a consistent framework that can be used to describe, assess and enhance implementation potential through identifying factors that promote and inhibit the routine incorporation of complex interventions into everyday practice [8, 9].

For example, our research team carried out a randomised controlled trial of an mhealth smartphone application to promote physical activity in primary care (SMART MOVE) which demonstrated a short term and sustained increase in physical activity in the intervention group [10]. The findings from such clinical research cannot change population health outcomes unless health care systems and professionals adopt them in practice [6]. The aim of this study was to conduct a theoretically informed analysis, using NPT, of the potential barriers and levers to the implementation of a mhealth intervention to promote physical activity in primary care.

## Methods

### SMART MOVE trial

SMART MOVE was an open label randomised controlled trial of a smartphone application to promote physical activity in primary care as part of an International Transnational Telemedicine Solutions (ITTS) project funded by the European Union Northern Periphery Programme [11]. The full study protocol has been published [12]. The trial intervention is summarised in Table 1. The trial demonstrated that the use of a smartphone application successfully promoted physical activity in primary care with a difference in mean improvement of 1029 (95% confidence interval 214 to 1843) steps per day favouring the intervention or approximately a half a mile [10].

**Table 1:** ‘SMART MOVE’ Randomised Controlled Trial

*‘SMART MOVE’ Trial*	
Registered with the International Standard Randomised Controlled Trials Register #ISRCTN99944116 and ethical approval obtained.	
90 Participants recruited by primary care health professionals or self-referred. Screened by the research team for inclusion suitability.	
Inclusion criteria: • Adult participants in the community • Over 16 years of age • Active android smartphone participants	Exclusion criteria: • Acute psychiatric illness • Pregnant women • Participants unable to undertake moderate exercise
At baseline screening meeting, participants were given study information, signed consent, were randomised and completed quality of life and mental health score questionnaires. BMI, blood pressure and heart rate were measured. Smartphone application Accupedo-Pro Pedometer was downloaded onto the smartphones but step count display was not made visible.	
Week 1: All participants continued their normal activity level while carrying the smartphone during all waking hours so the smartphone application could record their baseline step count while remaining invisible to the participant.	
End of week 1: Randomisation code broken and participants assigned to control or intervention groups.	
Control Group: • Smartphone application with step count continues to remain invisible • Given information on benefits of exercise • Instructed to increase physical activity with a goal of an additional 30 min walking exercise per day (equivalent to 10,000 steps per day)	Intervention Group: • Smartphone application and step count made visible (Fig. 1) • Given information on benefits of exercise • Instructed to increase physical activity with a goal of 10,000 steps per day and encouraged to use the smartphone application to achieve this goal
After completion of eight-week trial: Quality of life and mental health score questionnaire administered and BMI, blood pressure, heart rate recorded. Quantitative results were analysed with the Statistical Package for the Social Sciences (SPSS) for primary outcome of mean difference in daily step count between baseline and follow-up at eight weeks and also secondary outcomes. After trial completion, all control groups participants were then also shown how to use the App	
A qualitative evaluation was then carried out by interviewing a purposeful sample of post-trial participants to explore their experiences within four weeks of finishing the trial.	

This study reports a qualitative analysis that followed the trial describing a multi-perspective exploration of the barriers and levers to the implementation of this novel intervention amongst primary care service users and service providers. NPT, a theoretical sociological model from the implementation science domain was used to generate the topic guide, analyse the dataset and subsequently optimize the design of an implementation package to support the utilization and normalization of this novel exercise promotion tool into clinical practice. Ethical approval was obtained from the Clinical Research Ethics Committee at Merlin Park University Hospital, Galway.

### Sampling and recruitment

**Fig. 1:**
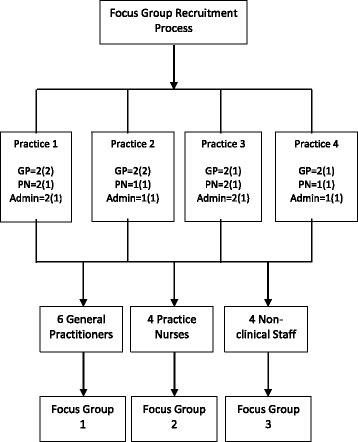
Recruitment of primary care service providers [*n* = number of staff in each practice (number recruited)]

The study took place in the West of Ireland with recruitment in the community from the Clare Primary Care Network within the Western Research and Education Network (WestREN), a network of over 180 family practices covering a mixed urban-rural population of approximately 500,000 patients which has been shown to nationally representative [13]. General practice in Ireland is highly computerised with most practices operating in a paperless fashion with a significant amount of coding of chronic disease but much less coding of each consultation [13]. In regard to primary care service providers, two practices that had, and two practices that had not, participated in the SMART MOVE trial were invited to take part and all consented to do so. Primary care service provider participants are described in Fig. 1. There were general practitioners (GP), practice nurses (PN) and non-clinical staff (NCS) from all four practices included in the study. Staff that had not participated in the trial were included with a view to enriching the discussions after being appraised of the subject matter to enhance their contribution.

As part of the SMART MOVE trial four primary care service users were recruited through purposeful sampling and semi-structured interviews [14] were employed to explore their experiences of using the trial intervention. This cohort of four participants, three from the intervention group and one from the control group were chosen for their capacity to describe in depth their experience of using the intervention, as well as representing diversity in terms of age, gender and control or intervention in the context of the trial (Table 2).

**Table 2:** Primary care service users interviewed

Participant code	Intervention or Control	Age range	Gender
P1	Intervention	50–60	F
P2	Intervention	50–60	M
P3	Intervention	30–40	F
P4	Control	60–70	F

### Data collection

The primary care service providers were invited to participate in focus groups [15] facilitated by the research team. An NPT generated topic guide (Table 3) was used to encourage discussion coherent with the study objectives. Open-ended questions were employed throughout so as not to bias how respondents might express themselves [16]. The format for the focus groups was as follows: (i) a presentation was delivered describing the SMART MOVE trial results and the associated qualitative evaluation of the trial [17] (ii) facilitated group discussion using the NPT generated topic guide and (iii) an open question and answer session. A refinement to the focus group method, the fishbowl technique [18] was employed.

**Table 3:** Focus group topic guide for primary care service providers

In the topic guide, the term “App” is used to refer to the smartphone application under study.	
Coherence is about sense making. 1. Did you have a clear understanding from the outset of how this intervention would benefit patients? 2. What strategies have you used previously to increase exercise amongst patients in the practice? 3. How does the App differ from previous strategies? 4. Do you think that one of your important professional roles is the discussion and promotion of increased activity levels with your clients/patients? 5. In your opinion, does the promotion of this strategy fit well with the overall goals and activity of your medical practice? Cognitive Participation is about engagement/‘buy in’: 1. How did you get involved and who told you about the idea? 2. Why was it right for you to get involved and promote this to your patient/clients? 3. Were you prepared to commit the time and effort to promote its use amongst patients/clients? 4. Who was driving this forward/encouraging involvement in this work? Collective action is about actions and interactions that are required to use the intervention: 1. Did the promotion of the App fit easily with your daily work practices or did you have to change your usual way of working/consulting with patients? 2. Can you talk about the training/education you received from the outset ….was this helpful/unhelpful and if so, in what ways? 3. What did you find easy and what did you find difficult about promoting the use of the App to your clients/patients? 4. Did you have to work with others to promote the use of the App – who? What was that experience like? Reflexive monitoring is about appraisal and evaluation: 1. Do you feel that using the App is an effective and worthwhile way to increase activity levels amongst patients and if so how do you know? 2. Can you tell about some of the feedback you’ve had from clients/patients who have used the App. 3. Do you have any suggestions for improving the promotion and use of the App amongst your clients/patients? 4. Is there anything you would do/do differently now in your consultations to promote the use of the application to patients?	

The one-on-one interviews were conducted by the research team with service users who had participated in the SMART MOVE trial. The questions were iterative and evolved accordingly throughout the interview process [19]. An NPT generated topic guide (Table 4) was used to encourage discussion coherent with the study objectives. The audio-taped focus group and individual interviews were professionally transcribed, following which the audio files and transcriptions were correlated as per the demands of reliable analysis [20]. All participants were consented for interview and for the use of anonymous quotations. Continuous observation of the concepts of saturation and sequential analysis appropriately steered the modification of both the individual interviews and the focus group discussions.

**Table 4:** Interview topic guide for service users

In the topic guide, the term “App” is used to refer to the smartphone application under study.	
Coherence: 1. Can I take you back to the ‘life before the trial (!)’ can you tell me about other strategies you have used in the past to increase your exercise 2. In what way was the idea of using the App different from these other strategies? Cognitive participation is about engagement/‘buy in’: 1. How did you get involved - who told you about the idea? 2. Can you tell me why you thought it was right for you to get involved in the trial and to give this App a go? Collective action is about actions and interactions that are required to use the intervention: 1. Now let’s focus on when you were starting to use the App itself, can you talk to me about starting to use it and the strategies that made it easy to do so and difficult to do so? 2. What was your sense of trust in the App as a device to support/stimulate your exercise routines? 3. Can you tell me about the different skills you needed to use the App? (develop some prompts here based on your knowledge of the different tasks that people had to perform to use the App) 4. What are your thoughts about using the App once/now that the trial is over? Reflexive monitoring is about appraisal and evaluation: 1. Was using the application an effective and worthwhile way to improve your exercise? 2. How do you know? 3. Is it worthwhile to keep using the App now that the trial is over?	

### Methodological considerations

Specific strategies to successfully address the challenges of implementation focused research include a preeminent role for theoretical approaches [5]. The Normalization Process Theory is one such theory from the field of implementation science offering researchers “a consistent framework that can be used to describe, evaluate and enhance the implementation potential of healthcare interventions*”* [1]. NPT offers a means of determining factors that help and hinder the successful implementation of interventions into daily practice. Moreover, it examines all stages of this process from early implementation to the point at which it becomes so completely ingrained into routine practice that it becomes ‘normalised’. It is important to note that this theory also describes how work practices can also become denormalised (i.e. use of paper notes in general practice) and equally normalisation can often be undesirable (i.e. over-utilisation of antibiotics in respiratory tract infections) [21].

Essentially the work that individuals and groups must undertake to enable an intervention to become normalised is the primary focus of NPT and comprises four main components [22, 23]. These four components are described below with examples from the current study given for illustration purposes:

i) *Coherence* is about sense making. How do stakeholders (primary care service providers whose role it is to promote and support the use of the smartphone application as well as the service users themselves who are the intended users of this novel exercise promotion tool) make sense of the intervention and consider its relevance to their lives?
ii) *Cognitive participation* is about engagement or ‘buy in’. Do stakeholders engage with this idea as a means of promoting behaviour change around exercise? For example what roles will service providers have to take on to facilitate or promote its implementation?
iii) *Collective action* is about actions and interactions that are demanded of both service providers and service users for the intervention to be promoted and utilized successfully. For example, do service providers report that promoting and supporting the use of the smartphone application amongst service users is overly burdensome in the context of their usual work demands?
iv) *Reflexive monitoring* is about appraisal and evaluation; how do service providers and service users formally and informally evaluate the use and benefits of the smartphone application in their lives and work environments respectively?

That NPT can act as a sensitizing tool allowing the researchers to scrutinize implementation issues pertaining to the use of the smartphone application in the particular context studied in the trial. This made it an ideal choice for the detailed examination of the implementation potential of this novel exercise promotion tool.

### Data analysis

Coding was undertaken by two researchers from different professional backgrounds (general practitioner and practice nurse). The data was reviewed periodically by the four member research team made of up of two general practitioners (including author) a nurse and a clinical engineer. QSR International NVivo 10 software [24] was employed as a data organisation tool to expedite the analysis and identification of relationships between coded ideas which facilitated understanding and evaluation of the interview information [25].

### Quality and rigour

In order to enhance the quality of data generation and analysis, coding was undertaken by two researchers from different professional backgrounds (general practitioner and practice nurse) to facilitate *inter-coder reliability* as well as generating increased reflexivity [26]. *Reflexivity* suggests an awareness of the means by which the researcher and the research process can shape the assembled data and includes the effects of the researcher’s personal characteristics and professional status [27] on the collected data and was further ensured by the exploration of themes by a four member research team made of up of two general practitioners (including author) a nurse and a clinical engineer [28]. *Fair dealing* describes the principle whereby the viewpoint of one group is never presented as if it expresses the sole truth about a situation. The research design specifically incorporated a range of different viewpoints which included those of general practitioners, practice nurses, non-clinical practice staff as well as service users [27].

## Results

As well as using the Normalisation Process Theory to generate the interviews and focus group topic guides for service providers and service users, it was also used to analyse the resulting dataset. Four themes emerged from the process of framework analysis. This process consists of five stages which included familiarization, thematic framework identification, indexing, charting, mapping and interpretation [29]. The themes were: personal and professional exercise strategies; roles and responsibilities to support active engagement; utilisation challenges; and evaluation, adoption and adherence.

### Theme 1: Strategies to promote exercise - personal and professional (sense-making work)

Learning about the smartphone application intervention describes the work service providers had to undertake to develop a complete understanding of this novel exercise promotion tool. It was immediately apparent that usually there was an absence of a formal strategy to promote increased physical activity amongst service providers.

*“No, again nothing formal, I suppose I might have you know outlined the benefits of weight bearing activities such as walking but I suppose nothing prescriptive or anything like that no.”* [Dr 3]

Both service providers and service users described having had no strategy previously to measure how much exercise they were doing nor did they know how much they needed to exercise to be considered active.

*“I was a bit shocked because I wasn’t doing as much running around as I thought I was doing.”* [P3]

Many service providers expressed exasperation that previous strategies were rarely successful despite genuine and repeated efforts to encourage the patients to increase their activity levels. Evidence of previous failed strategies were frequently cited. The fact that previous negotiations with service users were often preoccupied with the emotive issue of weight had rendered certain service providers much less inclined to discuss the topic of exercise in their routine work.

*“I think it’s a difficult thing to engage with patients, I’ve always found it, I’ve never been particularly comfortable around raising issues around exercise and weight....”* [Dr 1]

In comparison with previous negative experiences when promoting increased exercise to service users with weight problems, service providers who had utilised the smartphone application observed the manner in which promoting the use of the application allowed the focus of the conversation to shift largely towards exercise promotion rather than the weight issues of the individual service user. The novel idea of engaging with the smartphone technology and the step count screen widget and graphs to monitor their progress was a new concept to most, coupled with the convenience that they were already carrying the smartphone. This “depersonalisation” and neutral space offered by the technology seemed to help service providers in dealing with the sensitivities around issues of obesity and sedentary lifestyle and overlaps with theme 3 of collective action.

*“What was attractive about this was the sort of more neutral conversation you could have, …didn’t have to necessarily be associated with a conversation about them being obese, overweight or anything like that…..”* [Dr 1]

### Theme 2: Roles and responsibilities to support active engagement with the app (participation work)

Participants had definite expectations in regard to the technology altering their relationship with exercise and being a lever for change.

“*I hoped to ….incorporate fitness into my lifestyle really ….unless it’s the walk with the kids or something like that, (exercise) was incidental….So basically I wanted it to incorporate (the App) so I would have some exercise throughout, a regular exercise during the week and that I’d be able to continue it after the study, that was the aim.” [P3]*

Both service providers and service users described new motivation around behaviour change in relation to physical activity as a result of their introduction to, and experience with, this novel exercise promotion tool. It was of interest and well received by service users as it appeared to provide a clear structure for a strategy to increase their exercise levels to a point where they were confident that they were exercising enough to be considered active.

*“I think it is a good, you know to use because it’s quantifiable. You can see the amount of steps, it’s measurable. You know there's a definitive guide there.”* [PN2]

*“I think if you're going into a doctor’s surgery and it is a wonderful aid and it is a great awareness for someone, you know so you can say yes this is the goal of 10,000, you’re back here at 2,000 so in the next few weeks we’re going to try and see can you go from 2,000 to maybe 4,000 and 6,000, 8,000 up, give a step by step approach.”* [P3]

Service users provided numerous reasons for active engagement with this smartphone application as a means of increasing their activity levels. These included continual awareness of one’s step count in relation to daily step count goals through the instant feedback facilitated by the smartphone application visual settings which enabled goal setting and adjustment throughout the day.

*“I hated going out in the dark but I have gone out as far as the road and back to get my steps and I also had a, there’s a stepper upstairs and I went on that a few times…..My behaviour completely changed.”* [P1]

Certain service providers, particularly those from the two primary care medical centres that had not participated in the trial were initially apprehensive about promoting the intervention to service users owing to their own limited smartphone literacy. Understandably these service providers were also concerned that it would demand significant extra workload.

*“My apprehension is that people will come in for their smartphone you know to talk about but then they’ll start looking for medication and blood pressure and everything else along with it and then your consultation time is going to run over. So you know that’s one fear I have.”* [PN2]

In general, while service providers acknowledged its central importance, they frequently struggled with their exercise-promoting role as it often times made the service user uncomfortable.

*“I find a good barometer of the level of enthusiasm for this type of intervention is the haste with which the patient wants to get off that entire subject.”* [Dr 3]

Many of the service providers bought into the concept of it and started increasing their own physical activity and employed it to increase their activity levels.

*“We all, you know decided to engage with the process and I think it probably changed all our approach to the issues around weight and exercise….we’re all raising the topic a bit more and recording BMI… it has changed the way we operate as a team.”* [Dr 1]

Non-clinical staff didn’t see it as their role to promote the smartphone application directly to service users but suggested that they would be happy to assume a supporting role.

*“Maybe if a suggestion that a doctor or the nurse could make it known that if the patient had any problems that the admin staff are there to help…but it’s not your place to approach them.”* [NCS 2]

### Theme 3: Utilisation challenges (enacting work)

The actions and interactions required of service providers and service users to use the smartphone application were next considered. Service providers and service users both remarked that the simplicity of using a smartphone application was a significant lever to its utilisation.

*“You know which is nice, it’s very clear, like it’s a very accessible app to most people because there isn’t much vocabulary involved, there isn’t much text involved in it, it’s all very clear, so for that reason I think it’s very accessible to most people.”* [P3]

*“Anybody could use it…..if you could dial a number on a phone you could use it because it's on the screen and it just works.”* [PN2]

Primary care service providers, while doing the necessary work to familiarise themselves with the smartphone applications, remarked frequently on the significant motivational potential offered by the visual appeal and live feedback on step count. Moreover, they were impressed by the motivating influence of a formalized structure offered by step count and daily goals supported by continuous visual feedback in contrast to the relatively unstructured paradigm which characterized previous exercise advice.

*“Yeah, well like I’ve said they liked that they could set goals for themselves, they liked that they could look back and see what the previous day had, how they had done on previous days.”* [PN1]

Service users also remarked positively about the motivational potential of the smartphone application through its visual appeal and the immediacy of the step count feedback and felt confident that this was a clear advantage over previous strategies.

*“The daily graph because there was a kind of recognition of what you were doing a visual recognition of what you were doing.”* [P1]

However, both primary care service providers and service users reported challenges with the intervention, notably with respect to technological issues observed as a result of the smartphone application. However, in many instances these were quite readily overcome.

*“But the negative feedback as I said was all to do with the battery drainage.”* [PN2]

*“Well number one the battery (was) the biggest problem I had so I found myself then having to remember to keep charging it. I bought a charger for the car so that I could do it continuously….when it did get up and going and when you had access to it and were able to handle the technology yourself I found things did work better.” [P2]*

There were different views about trusting the accuracy of the App in recording physical activity but in general once service users became familiar with the technology, their levels of trust stabilised accordingly….

*“I think you have to realise that it is just an app and it is technology and technology isn’t going to be 100% the whole time and technology can be manipulated as well ….you just have to have a sense of honesty and trust in yourself, you know where you’re at and that’s it.” [P3]*

Service providers opined that now that the trial period was complete, role clarification should be central to the implementation phase. However, they felt that the promotion of the smartphone application fitted easily into their daily work practices and didn’t demand important changes to their usual routine or ways of working.

*“I think it’s simple…as you know a 30 second conversation…oh yeah we’re talking about exercise, there’s this really good app that I know about that counts your steps and gives you feedback on your exercise, would you be interested in it…ok go and have a conversation with the practice nurse about it…”* [Dr 1]

### Theme 4: Evaluation, adoption and adherence (reflexive monitoring)

Service users described in detail the increased sense of ownership and control they felt over their relationship with exercise. They also described how the App was a facilitator in this process of change and how the proximity of the technology on their smartphone made it very easy to connect and re-connect with this intervention.

*“I just think that because you have full control of it yourself its useful because lots of people don’t like to join clubs or they don’t like to join weight watchers, or they don’t like to join exercise programs or groups. But you have control of this yourself. And you can just do it privately and nobody need know anything about it. And you can motivate yourself with yourself and nobody else. It doesn’t cost anything, it’s completely free. And even if you fall off and stop working at it today you can always start a month down the line or three weeks down the line or whenever. You can re-start just get yourself into gear and get going again.” [P4]*

Service users also described the App as an educator which increased their awareness of their exercise patterns. In addition, participants differed in their longer term use of the App with some just using it to stimulate behaviour change while others continued to require the motivational effects of the technology.

*“I don’t carry it around with me all the time but I’m more aware now, ok I have a sense of right I know roughly how many steps I have now and I really need to go for a walk, or I really need to go for a long walk this evening, you know that type of way.” [P3]*

*“I have to say that that app has… it’s kind of changed me…..I don’t know how I’d be if I turned it off. I won’t turn it off, not for the moment anyway. I am quite happy to have it on because I am much more conscious of going out for that walk.” [P1]*

Service providers remarked that they were determined to focus on simplifying the process when implementing the smartphone application into routine practice. Moreover, service providers who hadn’t been involved in the trial expressed a readiness to utilise this novel exercise promotion tool in routine practice.

*“Well I think the way has been simplified …… the best idea is to keep it simple…it’s not a magic pill, it’s just something extra to have, another conversation you can have, it’s another little prod that you can give in the right direction…non-judgmental way, provides a neutral space to talk about the issue…I think it’s just something extra.”* [Dr1]

*“I suppose the confidence that working with it has brought, you know…you feel a bit more enthusiastic and you feel confident about being able to talk to patients about it.”* [PN 1]

A cascade effect (defined as an unforeseen series of events which can occur due to an action affecting a particular environment) described in previous research on smartphone interventions [17] was also noted. The smartphone application appeared to arouse curiosity and encouraged others to initiate or become involved in other physical activity interventions.

*“We saw effects in the community in terms of a walking group and running group that was formed by somebody who was involved, who downloaded the application.”* [Dr 1]

Primary care service providers offered a number of ideas to promote increased uptake of the smartphone application as an exercise promotion tool.

The need for a specific service provider staff member to drive the utilisation of the smartphone application by other team members was felt to be an essential ingredient in ensuring successful implementation. That all service providers promoting it should have the application initiated on their own smartphones and ideally having experience of utilising it to change their own exercise behaviour was suggested as an additional lever that may increase uptake.

*“I think the last thing is that the person who is spearheading the whole thing has to kind of lead by example themselves.”* [Dr 4]

## Discussion

### Summary

This qualitative evaluation of the SMART MOVE Trial demonstrates that introducing a new healthcare intervention into an Irish primary health care context demands a comprehensive evaluation of not only the intervention itself but also of the environment in which it is to operate. However, it is evident that despite certain obstacles the opportunity exists for the successful implementation of a novel healthcare intervention. For this to be successful, it must address a hitherto unresolved healthcare need (*structured and measurable strategy to increase activity levels)* in a particular context *(Irish primary health care),* that has strong usability attributes for both disseminators *(primary care service providers)* and target users *(primary care service users)* and coheres strongly with the core objectives and culture of the health care environment in which it is to operate.

### Comparison with existing literature

The potential barriers and levers to the implementation of this new healthcare intervention in a primary healthcare context, as identified by examination of the dataset, bear many similarities to those identified in existing literature. In addition, theyhave critically influenced the design of the implementation package, which is in effect a guideline to support the utilisation of the smartphone application by primary care service providers to promote increased activity amongst primary care service users. The decision to utilise the Normalisation Process Theory to evaluate and optimize the implementation potential of this novel exercise promotion tool is strongly reflected in the literature where it is evident that it has already been extensively used to develop and evaluate complex health care interventions [30, 31]. The data generated in this study illustrates the importance of reflecting on the numerous levels at which healthcare is delivered, their respective cultural contexts and how they interplay which is increasingly recognized as guiding precept of implementation research [32].

For this study specific benefits were that NPT was used to generate the focus group and interview topic guides and to analyse the resulting dataset allowed us to identify factors that were likely to promote and inhibit the incorporation of this novel exercise promotion tool into an Irish primary health care environment. As this exercise promotion tool is entirely novel and although the existing literature was therefore unable to fully encompass the experience of service providers and service users in their efforts to utilise this smartphone application as an exercise promotion tool, an important number of similarities with successful implementation experiences elsewhere were identified.

Once technical issues had been addressed, simplicity and ease of use characterised the experience of most disseminators (service providers) and target users (service users) of the smartphone application in this study which is a noted characteristic of health care interventions that have been successfully normalised into routine practice [33]. That the trial provided an evidence base for the effectiveness of the smartphone application in promoting increased activity levels was an additional attribute that was observed to increased implementation potential in other studies [34]. The fact that the implementation package design has been exclusively shaped by primary care service providers and primary care service users who constitute the target group of the implementation strategy is an attribute known to amplify implementation potential [35]. Moreover, promotion of the smartphone application by primary care service providers to potential users is a process that is closely allied to clinical decision making which has been identified as an lever to implementation elsewhere [36]. Negative attitudes from superiors along with poor support reduces implementation while conversely the presence of an identifiable staff member to act as a peer advisor and driver of utilization increases implementation potential [30, 34] and these experiences were also reflected in our results.

### Strengths and limitations

The study strengths included the sampling framework which included a balance of views from all stakeholders, namely: primary care service providers including clinical and administration staff along with primary care service users. Within the focus groups the views of those who had experience of the trial in addition to contributions from a wider cohort of primary care service providers that had not yet used the intervention enriched the focus group discussions.

Moreover, the focus groups, which utilised the fishbowl method and were divided along professional lines was deliberately designed with a view to enhance data generation. The use of the Normalisation Process Theory to generate the focus group and interview topic guides and to frame the data analysis was an additional strength, offering as it does a theoretical approach to determining factors that help and hinder the successful implementation of complex interventions into daily practice. The study limitations were the small numbers of service users and the homogenous cultural characteristics of the sample being all Irish Caucasian clinicians, administration staff and participants, which potentially may not be generalizable to other contexts and populations.

### Implications for practice

Primary health care service providers, exasperated by the repeated failure of previous attempts to promote exercise, identified certain key attributes of this novel exercise promotion tool that are likely to positively impact its implementation into routine practice. The smartphone application offered a clearly defined and quantifiable plan to increase exercise versus previous unstructured approaches, a strong motivational potential afforded by the continuous visual feedback and strong usability qualities for both primary care service providers and service users. In conjunction with increasing smartphone literacy and growing market penetrance of smartphone devices along with its coherence with the overall goals of the healthcare environment in which it is to be implemented, this solidly positions this novel exercise promotion tool for future implementation. However, implementation challenges do exist including technical concerns such as battery drainage, limited smartphone literacy and ownership amongst elderly populations and incomplete compatibility with iPhone devices. Service providers concerns included having sufficient smartphone literacy to allow effective promotion of the smartphone application, imposition on existing work routines, requirement for a staff member to drive utilization and wider issues such as on-going reductions in funding of the primary health care sector.

## Conclusions

We are aware that problematic translational gaps continue to exist between demonstrating the positive impact of healthcare interventions and the implementation and normalisation of such interventions into routine daily practice. Following on from this study, an implementation package will be developed for rollout to primary care using the NPT generated dataset and analysis to inform this process. We believe that the process used to develop this implementation package amplifies the implementation potential of such an intervention in primary care.

## Abbreviations

GP: General practitioner
ITTS: International transnational telemedicine solutions
NCS: Non-clinical staff
NPT: Normalisation process theory
PN: Practice nurse
WestREN: Western research and education network
